# Advances in GLP-1 treatment: focus on oral semaglutide

**DOI:** 10.1186/s13098-021-00713-9

**Published:** 2021-09-15

**Authors:** Freddy G. Eliaschewitz, Luis Henrique Canani

**Affiliations:** 1 CPClin/DASA Clinical Research Center, Avenida Angélica, 2162, São Paulo, CEP 01228-200 Brazil; 2 Endocrinology Division of Hospital de Clínicas de Porto Alegre and Department of Internal Medicine, Medical School of Federal, University of Rio Grande Do Sul, Porto Alegre, Brazil

**Keywords:** Oral semaglutide, Glycemic control, GLP-1 RA, PIONEER program, Type 2 diabetes

## Abstract

**Background:**

There is currently a large arsenal of antidiabetic drugs available to treat type 2 diabetes (T2D). However, this is a serious chronic disease that affects millions of adults worldwide and is responsible for severe complications, comorbidities, and low quality of life when uncontrolled due mainly to delays in initiating treatment or inadequate therapy. This review article aims to clarify the therapeutic role of the oral formulation of the glucagon-like peptide 1 receptor agonist (GLP-1 RA) semaglutide in treating typical T2D patients. The discussion focused on metabolic, glycemic, and weight alteration effects and the safety of the therapy with this drug.

**Main text:**

Therapy with glucagon-like peptide 1 receptor agonist (GLP-1 RA) promotes strategic changes in the pathophysiological pathway of T2D and improves the secretion of glucagon and insulin, which results in a reduction in blood glucose levels and the promotion of weight loss. Until recently, the only route for semaglutide administration was parenteral. However, an oral formulation of GLP-1 RA was recently developed and approved by the Brazilian Health Regulatory Agency (ANVISA) and the Food and Drug Administration (FDA) based on the Peptide Innovation for Early Diabetes Treatment (PIONEER) program results. A sequence of 10 clinical studies compared oral semaglutide with placebo or active standard-of-care medications (empagliflozin 25 mg, sitagliptin 100 mg, or liraglutide 1.8 mg) in different T2D populations.

**Conclusions:**

Oral semaglutide effectively reduces glycated hemoglobin (HbA1c) levels and body weight in a broad spectrum of patients with T2D and shows cardiovascular safety. Oral semaglutide broadens therapy options and facilitates the adoption of earlier GLP-1 RA treatment once T2D patients present low rates of treatment discontinuation. The main adverse events reported were related to the gastrointestinal tract, common to GLP-1 RA class drugs.

## Background

Diabetes mellitus is a major public health problem worldwide, as 10% of adults have the disease, which corresponded to 463 million individuals in the world in 2019 [1]. By 2045, this number will rise to 700 million [1]. Type 2 diabetes (T2D) corresponds to 90% of the cases, but 50.1% of these individuals did not have timely access to the diagnosis or appropriate treatment [1]. Moreover, 374 million adults have insulin resistance, presenting a high risk for T2D [1]. The prevalence of T2D is rapidly increasing in three regions of the world: South America, Asia, and Eastern Europe [1]. Brazil is one of the 10 countries with the highest number of patients with diabetes mellitus [2], with a prevalence of 11.9% [3], accounting for 12.5 million individuals in 2017 and 20.3 million by the year 2045 [2].

T2D is currently one of the top 10 causes of death worldwide; in addition to affecting the elderly, this disease continues to heavily affect patients under 60 years old and has an important economic impact: in 2019, the health costs of T2D reached $760 billion in the world [1]. T2D is commonly associated with relevant clinical conditions that are implicated in worse prognosis, increased mortality, and negative impacts on patient health-related quality of life, such as obesity and cardiovascular disease (CVD) [4]. The prevalence of obesity in patients with T2D is three times higher than that observed in the general population [5]. In Brazil, 75% of T2D patients are beyond the appropriate body mass index (BMI), and 30% have obesity [5]. Obesity increases the risk of death in patients with T2D, with an estimated 20% increase in mortality risk for each 5 kg/m^2^ increase in BMI [6]. Furthermore, this association has a direct linear relationship, with the lowest mortality risk described among patients with BMI equal to 22.5–24.9 kg/m^2^ [6]. Weight loss with diet and physical activity can prevent or reverse T2D [7]. The DIRECT trial demonstrates that a weight loss of approximately 15 kg can lead to T2D remission in approximately 80% of patients with obesity and T2D [8]. However, most drug classes involved in T2D therapy promote weight gain (insulin, sulfonylureas, and others) [9]. Therefore, it is important that antihyperglycemic therapies do not increase weight and ideally promote weight loss [9, 10].

CVD is considered the leading cause of death in patients with T2D [11]. The results from a global review that evaluated 57 worldwide articles showed that CVD was responsible for 50.3% (95% CI 37.0–63.7%) of all deaths in patients with T2D [11]. Globally, 32.2% of these patients had some form of CVD from 2007 to 2017 [11]. More recently, the CAPTURE study estimated that the prevalence of established CVD in nearly 10,000 adults with T2D across 13 countries was 34.8% (95% CI 32.7–36.8%) [12]. In Brazil, CAPTURE estimated that the CVD prevalence in T2D patients was 43.9% (95% CI 40.9–46.8%) [12]. More specifically, 37.6% (95% CI 34.7–40.5%) had atherosclerotic CVD, 27.9% (95% CI 25.2–30.5%) had coronary heart disease, 12.4% (95% CI 10.4–14.4%) had heart failure, 8.7% (95% CI 6.8–10.5%) had cerebrovascular disease, and 3.4% (95% CI 2.3–4.5%) had carotid artery disease [12].

Controlling blood sugar is an extremely important practice to avoid or delay chronic diabetic complications [13]. According to the United Kingdom Prospective Diabetes Study (UKPDS), a decrease of 1% in glycated hemoglobin (HbA1c) results in a 21% reduction in the risk of any endpoint related to diabetes, 21% in the risk of death related to diabetes, 14% in the risk of myocardial infarction, and 37% in the risk of microvascular complications [13]. Notwithstanding the efforts to improve the treatment of T2D with the development of new drugs, the percentage of patients achieving optimal glycemic control (HbA1c  <  7.0%) is still lower than desired, and mortality remains high, indicating the necessity of new therapies for T2D [14]. The global perspective DISCOVER study program demonstrated that exists a failure to monitor blood glucose in T2D patients that results in poor levels of glycemic control at initiation of second line therapy, particularly in lower-middle- and upper-middle-income countries [15]. At this stage of treatment, 26.7% of the tested patients had an HbA1c level  ≥  9.0% [15]. Lack of efficiency was the most stated reason for choosing a second-line therapy, followed by physician preference, patient request, and side effects [15]. T2D can be treated and managed by healthy eating, regular physical activity, and medications to lower blood glucose levels [16]. Insulin is also commonly used to control blood glucose in this cohort of individuals [16]. However, more recent treatments, including the glucagon-like peptide 1 receptor agonist (GLP-1 RA) class, are effective and have other protective benefits, such as CV safety, but are injectable [16].

This review article is about the therapeutic role of the oral formulation of the GLP-1 RA semaglutide in treating T2D based on the results obtained in the Peptide Innovation for Early Diabetes Treatment (PIONEER) program.

## The challenges of an oral peptide

Peptides are short amino acid monomer chains that are smaller than 100 amino acids, placing them between small molecules and proteins [17]. The advantages of using a peptide as a potential therapeutic drug are numerous, with highlights including the broad range of targets, low toxicity, high chemical and biological diversity, high potency and selectivity, good efficacy, safety, tolerability, low accumulation in tissues, and standard synthetic protocols [18]. However, experts face several limitations during the development process of a peptide, including limited oral bioavailability, elevated production costs, short half-life, rapid clearance, low metabolic stability, poor membrane permeability, and a tendency for aggregation, and some peptides can have immunogenic sequences [18].

The development of multiple strategies allowed an increase in the absorption of administered peptide medications [19]. These approaches include (1) permeation enhancers that can target either the transcellular route or the paracellular route; (2) modulation of pH; (3) direct enzyme inhibition; (4) peptide cyclization that removes exposed N and C termini from peptides and reduces the cleavage susceptibility by enzymes; (5) mucus-penetrating agents to enhance the rate of passage of peptides across the mucus barrier; (6) cell-penetrating peptides that interact with membrane glycosaminoglycans and deliver the peptide via exocytosis; (7) intestinal patches that physically protect a small reservoir of drug from local degradation while positioning the drug close to the absorptive epithelium; (8) hydrogels that facilitate prolonged retention and enable a prolonged peptide residency time within specific gut regions while simultaneously resisting enzymatic degradation; and (9) microneedle devices and milliposts that are important to minimize the effect of protein size on bioavailability [19].

Some preclinical trials successfully developed oral peptides; however, the clinical application did not show sufficient bioavailability, and the technology used needed to be improved [19, 20]. Insulin provides an example of some clinical trials that evaluated the efficacy and safety of a rapid-acting oral insulin formulation as well as all the failures [21]. The trials tested distinct technical approaches, different targets (stomach, small intestine, or colon), and a wide range of drug delivery systems [21]. However, problems such as inter- or intraindividual variability, gastric emptying rate, food ingestion timing, and the risk of differences in pharmacodynamic responses potentially enhanced the likelihood of hypoglycemia [21].

## Development of oral semaglutide: a GLP-1 ra peptide

GLP-1 is an intestinal hormone of the incretin family responsible for amplifying insulin secretion, suppressing glucagon release, delaying gastric emptying, and decreasing insulin resistance [19, 22]. In the central nervous system, GLP-1 acts as a neurotransmitter responsible for signaling satiety via the brainstem-hypothalamus [23–25]. Peripherally, GLP-1 reduces energy intake and affects all components of appetite regulation: increased satiety and fullness and decreased hunger and prospective food consumption [26, 27]. Together, these effects promote physiological satiety [28, 29].

GLP-1 RA is an effective treatment option for T2D but was available only in parenteral formulations since peptide-based drugs, including GLP-1 RA, have very low bioavailability when administered orally [30, 31]. Semaglutide is a GLP-1 RA that can be used subcutaneously once a week as a treatment for T2D [30, 31]. This injectable molecule is a potent and long-acting GLP-1 analog with 94% homology with human native GLP-1 [32]. Three key structural differences provide the extended pharmacokinetics of this drug, namely, the substitution of Ala with Aib at position 8 that increases enzymatic (DPP4) stability, attachment of a linker and C18 di-acid chain at position 26 that provides strong binding to albumin and substitution of Lys with Arg at position 34 that prevents C18 fatty acid-binding at the wrong site [32]. The phase 3 clinical trial program (SUSTAIN) compared subcutaneous semaglutide to placebo or other active standard-of-care medications in patients with T2D [30, 31]. These trials showed that the subcutaneous semaglutide group had a greater reduction in HbA1c and body weight loss rates and that the semaglutide had a well-characterized safety profile [30, 31]. However, T2D requires long-term treatment, and in general, an oral formulation is preferred by patients and could improve medication adherence. [19]. The oral formulation of semaglutide was approved on September 20th, 2019, by the Food and Drug Administration (FDA) and on October 20th, 2020, by the Brazilian Health Regulatory Agency (ANVISA) to treat T2D based on the PIONEER program, a sequence of phase III clinical trials showing the efficacy and safety of oral semaglutide in comparison with placebo or other active standard-of-care medications [33, 34].

The coformulation of semaglutide with sodium N-[8-(2-hydroxybenzoyl) amino] caprylate (SNAC), a transcellular permeation enhancer, ensured the bioavailability of the oral formulation [19]. In association with semaglutide, SNAC has a predominantly transcellular transit mode via the gastric epithelium, where absorption occurs 60–140 min after ingesting a tablet containing 10 mg of semaglutide and 300 mg of SNAC in humans and dogs [19]. SNAC probably also attenuates semaglutide enzymatic digestion as it increases the local gastric pH [19].

Phase I clinical trials showed that the concomitant use of contraceptive pills, omeprazole, or other drugs commonly prescribed for T2D, as well as renal failure (any degree), do not compromise oral semaglutide pharmacokinetics [35–37]. To ensure bioavailability, patients should take oral semaglutide in the morning in the fasting state with up to half a glass of water (approximately 120 mL), at least 30 min before the ingestion of any food or other medications [19]. Patients should gradually increase the dose of oral semaglutide to minimize the risk of GI adverse events, starting with 3 mg once daily and, after 30 days, increasing to 7 mg and then, if necessary, to 14 mg once daily [19].

## Design of the pioneer trials

Based on a phase II clinical trial [38], a sequence of eight multicenter, randomized phase III clinical trials, the PIONEER program, assessed the efficacy and safety of oral semaglutide [39–46]. Together, the trials evaluated oral semaglutide in 6163 patients who were at least 18 years old, had a diagnosis of T2D for at least 90 days before screening, and, for most patients, had inadequate glycemic control [39–46]. These trials included patients at the beginning of treatment (PIONEER 1), patients with advanced disease (PIONEER 8), and special populations (PIONEER trials 5 and 6). Furthermore, the trials also evaluated oral semaglutide versus oral antidiabetic drugs (OADs) (PIONEER trials 2 and 3) or the GLP-1 RA liraglutide (PIONEER 4) and oral semaglutide in flexible doses (PIONEER 7) (Table 1) [39–46].

**Table 1:** Summary of design and primary endpoints across the PIONEER trials considering trial product without rescue medication (trial product estimand)

Trial	Population^a^	Primary endpoint	Treatment	Results primary endpoint (%)	EDT (95% CI)	p
PIONEER 1	703 adults with T2D uncontrolled with diet and exercise	Change in HbA1c from baseline to week 26	Sema 3 mg	− 0.8	− 0.7% (− 0.9 to − 0.5)	< 0.001
			Sema 7 mg	− 1.3	− 1.2% (− 1.5 to − 1.0)	< 0.001
			Sema 14 mg	− 1.5	− 1.4% (− 1.7 to − 1.2)	< 0.001
			Pbo	− 0.1	–	–
PIONEER 2	822 adults with T2D uncontrolled on metformin	Change in HbA1c from baseline to week 26	Sema 14 mg	− 1.4	− 0.5% (− 0.7 to − 0.4)	< 0.0001
			Empa 25 mg	− 0.9	–	–
PIONEER 3	1864 adults with T2D uncontrolled with metformin and/or sulfonylurea	Change in HbA1c from baseline to week 26	Sema 3 mg	− 0.5	0.2% (0.1–0.4)	< 0.001
			Sema 7 mg	− 1.1	− 0.3% (− 0.4 to − 0.2)	< 0.001
			Sema 14 mg	− 1.4	− 0.6% (− 0.7 to − 0.5)	< 0.001
			Sita 100 mg	− 0.8	–	–
PIONEER 4	711 adults with T2D on metformin with or without an SGLT2 inhibitor	Change in HbA1c from baseline to week 26	Sema 14 mg	− 1.3	− 0.2% (− 0.3 to − 0.1)^b^	0.0056^b^
					− 1.2% (− 1.4 to − 1.0)^c^	< 0.001^c^
			Lira 1.8 mg	− 1.1	–	–
			Pbo	− 0.1	–	–
PIONEER 5	324 adults with T2D and moderate renal impairment on metformin and/or sulfonylurea, or basal insulin	Change in HbA1c from baseline to week 26	Sema 14 mg	− 1.1	1.0% (− 1.2 to − 0.8)	< 0.0001
			Pbo	− 0.1	–	–
PIONEER 7	504 adults with T2D inadequately controlled on one of two oral glucose-lowering drugs	Proportion of patients with HbA1c less than 7.0% at week 52	Sema flex	63	–	< 0.0001
			Sita 100 mg	28	–	–
PIONEER 8	731 adults with T2D under insulin therapy with or without metformin	Change in HbA1c from baseline to week 26	Sema 3 mg	− 0.6	− 0.6 (− 0.7 to − 0.4)	< 0.0001
			Sema 7 mg	− 1.0	− 1.0 (− 1.2 to − 0.8)	< 0.0001
			Sema 14 mg	− 1.4	− 1.4 (− 1.6 to − 1.2)	< 0.0001
			Pbo	− 0.0	–	–

*CI* confidence interval; *Dula* dulaglutide; *EDT* estimated treatment differences; *Empa* empagliflozin; *Flex* flexible dose (3, 7 or 14 mg); *Lira* liraglutide; *Pbo* placebo; *Sema* oral semaglutide; *Sita* sitagliptin; *T2D* type 2 diabetes

^a^Randomized

^b^Versus liraglutide

^c^Versus placebo

In all PIONEER trials, typical T2D patients received gradually increased doses of oral semaglutide as described earlier, except for PIONEER 7, in which the medication dose started at 3 mg and, after an 8-week interval, could be increased or decreased depending on the patient’s glycemic response and gastrointestinal tolerability (flexible dose-adjustment approach) [39, 40, 42–46].

PIONEER trials 1, 5, and 8 evaluated the effects of oral semaglutide in comparison with placebo [39, 43, 46]. PIONEER 2 compared oral semaglutide with empagliflozin 25 mg, PIONEER trials 3 and 7 with sitagliptin 100 mg, and PIONEER 4 with placebo and liraglutide 1.8 mg [40–42, 45].

The PIONEER 5 trial evaluated patients with advanced disease (T2D mean duration of 14.0 years and taking metformin and/or sulfonylurea or insulin with or without metformin) [43]. Specifically, this trial intended to explore the efficacy and safety of oral semaglutide 14 mg compared with placebo in individuals with T2D and moderate renal impairment [estimated glomerular filtration rate (eGFR), 30–59 mL/min/1.73 m^2^] [43]. Finally, the design of PIONEER 6 assessed cardiovascular (CV) outcomes of oral semaglutide treatment versus placebo in patients with T2D at high CV risk [age  ≥  50 years with established CV or chronic kidney disease (CKD), or age  ≥  60 years with CV risk factors only] [44].

All PIONEER trials verified HbA1c and weight [39–46]. PIONEER trials 1–5, 7, and 8 aimed to evaluate glycemic control as the primary endpoint and weight as the secondary endpoint [45]. On the other hand, the primary endpoint of PIONEER 6 was the time from randomization to the first occurrence of a major adverse CV event (MACE) [44]. The secondary endpoints included time from randomization to the first occurrence of an expanded composite CV endpoint; time from randomization to the first occurrence of the individual components of the expanded composite CV endpoint; time to the first occurrence of a composite of all‐cause death, non‐fatal myocardial infarction or non‐fatal stroke; time to all‐cause death; time to permanent trial drug discontinuation due to adverse events; the number of serious adverse events; and changes from baseline in a variety of laboratory and clinical assessments [44].

## Glycemic control in the pioneer program

In PIONEER 1, in patients with early T2D and management only by diet and exercise, once-daily oral semaglutide monotherapy at doses of 7 and 14 mg significantly reduced HbA1c levels compared to placebo at week 26 [estimated treatment difference (ETD) − 1.2%, 95% confidence interval (CI) − 1.5 to − 1.0 (7 mg); − 1.4%, 95% CI − 1.7 to − 1.2 (14 mg); p  <  0.001 for both] [39]. Furthermore, the use of oral semaglutide suggests improvement in pancreatic insulin secretion assessed through homeostasis model assessment of β-cell function (HOMA-β) [ETD 1.63%, 95% CI 1.44–1.85 (7 mg), 1.71%, 95% CI 1.51–1.93 (14 mg); p  <  0.001 for both] [39]. The fasting C-peptide level represents the insulin that the pancreas itself produces and was also increased in the semaglutide group compared to the placebo group [ETD 1.11 ng/ml, 95% CI 1.02–1.20; p  =  0.01 (7 mg), 1.05 ng/ml, 95% CI 0.97–1.14; p  =  0.19 (14 mg)] [39, 47].

In PIONEER 4, the results were similar in that oral semaglutide 14 mg treatment, in association with metformin with or without an SGLT2i, was superior to placebo in reducing HbA1c at week 26 (ETD − 1.2%, 95% CI − 1.4 to − 1.0; p  <  0.001) [42]. In this trial, oral semaglutide 14 mg had significantly greater decreases in HbA1c than both subcutaneous liraglutide (ETD − 0.2%, 95% CI − 0.3 to − 0.1; p  =  0.0056) and placebo (ETD − 1.2%, 95% CI − 1.4 to − 1.0; p  <  0.0001) at week 26 [42].

In PIONEER 2, oral semaglutide 14 mg treatment resulted in superior changes in HbA1c from baseline at week 26 in patients with T2D uncontrolled with metformin compared with empagliflozin 25 mg (ETD − 0.5%, 95% CI − 0.7 to − 0.4; p  <  0.0001) [40]. In this trial, the HOMA-β index was higher at week 26 in the oral semaglutide group than in the empagliflozin 25 mg group (ETD 1.50%, 95% CI 1.39–1.62; p  <  0.0001) [40]. The doses of 7 and 14 mg of oral semaglutide administered to patients with T2D uncontrolled with metformin and/or sulfonylurea in PIONEER 3 were both superior to sitagliptin 100 mg in decreasing HbA1c levels at week 26 [ETD − 0.3%, 95% CI − 0.4 to − 0.2 (7 mg), − 0.6%, 95% CI − 0.7 to − 0.5 (14 mg); p  <  0.001 for both] [41].

In PIONEER 7, the proportion of patients achieving an HbA1c target of less than 7.0% (53 mmol/mol) at week 52 was 63% using oral semaglutide with a flexible-dose adjustment strategy compared with 28% using sitagliptin 100 mg (p  <  0.0001) [45]. Oral semaglutide resulted in significantly greater decreases in HbA1c than sitagliptin 100 mg did at week 52 (ETD, − 0.7%; 95% CI − 0.9 to − 0.5; p  <  0.0001) [45].

PIONEER 5 included patients with moderate renal impairment taking metformin and/or sulfonylurea or insulin with or without metformin [43]. The study population was older than the other trials (70 years on average) [43]. In this population, 14 mg of oral semaglutide was significantly more effective than placebo in reducing HbA1c levels at week 26 (ETD, − 1.0%; 95% CI − 1.2 to − 0.8; p  <  0.0001) [43].

In PIONEER 8, patients with advanced disease (T2D mean duration of 15.0 years and taking insulin with or without metformin) had significant reductions in HbA1c levels with both the 7 mg dose and the 14 mg dose compared with the placebo at week 26 [ETD − 1.0%, 95% CI − 1.2 to − 0.8 (7 mg), − 1.4%, 95% CI − 1.6 to − 1.2 (14 mg); p  <  0.0001 for both]. The observed effect was maintained until week 52 [ETD − 0.9%, 95% CI − 1.1 to − 0.6 (7 mg), − 1.3%, 95% CI − 1.5 to −1.0 (14 mg); p  <  0.0001 for both] [46]. Additionally, at baseline, the overall mean total daily insulin dosage was 58 units [46]. During the 8-week study drug initiation period, a 20% reduction in this dosage was recommended when initiating oral semaglutide, with the majority (75.3%) of patients having their insulin dosage reduced by 15–25%, 8.4% with dose reduction  <  15% and 3.4% with a  >  25% reduction [46]. At week 26, patients using 7 mg and 14 mg of oral semaglutide reduced their insulin dosage when compared to placebo [ETD − 3 units, 95% CI − 6 to − 1; p  =  0.0169 (7 mg), − 6 units, 95% CI − 8 to − 3; p  <  0.0001 (14 mg)] [46]. The observed effect was maintained until week 52 (ETD − 8 units, 95% CI − 11 to − 4 (7 mg), − 12 units, 95% CI − 15 to − 8 (14 mg); p  <  0.0001 for both] [46].

Table 1 is a compilation of all the above data, and it considers the trial product as an estimand in evaluating the effect of oral semaglutide compared with placebo without the confounding effect of rescue medication. In other words, this estimand reflects the treatment effect for all randomized patients (trial product estimand).

## Weight loss in the pioneer program

Independent of the T2D background of the patient, the PIONEER program showed that oral semaglutide treatment effectively reduced body weight compared with placebo or other active standard-of-care medications [39–43, 45, 46]. In PIONEER 4, patients with uncontrolled T2D taking metformin with or without an SGLT2i and treated with 14 mg of oral semaglutide had a higher weight loss in the program compared with placebo at week 26 (ETD, − 4.0 kg; 95% CI − 4.8 to − 3.2; p  <  0.0001), and this reduction was sustained at week 52 (ETD, − 3.8 kg; 95% CI − 4.8 to − 2.7; p  <  0.0001). In this trial, 14 mg of oral semaglutide treatment also provided a pronounced body weight reduction compared to the GLP-1 RA liraglutide 1.8 mg at week 26 (ETD, − 1.5 kg; 95% CI − 2.2 to − 0.9; p  <  0.0001) and week 52 (ETD, − 1.8 kg; 95% CI − 2.6 to − 1.0; p  <  0.0001).

Compared with placebo, oral semaglutide was more effective in reducing body weight in PIONEER 1 at week 26 [ETD − 1.0 kg, 95% CI − 1.8 to − 0.2; p  =  0.01 (7 mg), − 2.6 kg, 95% CI − 3.4 to − 1.8; p  <  0.001 (14 mg)], PIONEER 5 even with patients taking metformin and/or sulfonylurea or insulin with or without metformin [ETD − 2.7 kg, 95% CI − 3.5 to − 1.9; p  =  0.0001 (14 mg)] and PIONEER 8 regardless of the background insulin at week 26 (ETD − 2.5 kg, 95% CI − 3.2 to − 1.8 (7 mg), − 3.7 kg, 95% CI − 4.4 to − 3.0 (14 mg); p  <  0.001 for both] [39, 43, 46]. This efficacy in PIONEER 8 was long-lasting and seen at week 52 [ETD − 3.5 kg, 95% CI − 4.5 to − 2.6 (7 mg), − 4.9 kg, 95% CI − 5.9 to − 3.9 (14 mg); p  <  0.0001 for both] [46]. In this study, the authors highlighted that the significant reduction in weight concerning the placebo indicated that oral semaglutide might help overcome some side effects caused by insulin [46].

Compared with sitagliptin 100 mg, oral semaglutide was associated, in PIONEER 3, with significantly pronounced reductions in body weight with 7 mg and 14 mg doses at week 26 [ETD − 1.5 kg, 95% CI − 2.0 to − 1.1 (7 mg), − 2.6 kg, 95% CI − 3.1 to − 2.1 (14 mg); p  <  0.001 for both] and week 52 [ETD − 1.5 kg, 95% CI − 2.1 to − 0.9 (7 mg), − 2.9 kg, 95% CI − 3.5 to − 2.3 (14 mg); p  <  0.001 for both] [41]. After 52 weeks, a flexible dose of oral semaglutide also showed greater reductions in body weight in comparison with sitagliptin 100 mg in PIONEER 7 (ETD − 2.2 kg, 95% CI − 2.9 to − 1.5; p  <  0.0001) [45]. In patients receiving background metformin in PIONEER 2, oral semaglutide 14 mg showed no inferiority against empagliflozin 25 mg in reducing body weight at week 26 (ETD − 0.4 kg, 95% CI − 1.0 to − 0.1; p  =  0.1358) [40]. However, 14 mg of oral semaglutide achieved a significantly greater body weight reduction than 25 mg of empagliflozin at week 52 (ETD − 0.9 kg, 95% CI − 1.6 to − 0.2; p  =  0.0114) [40]. In this trial, patients answered a questionnaire adapted from the Food Craving Record that showed that patients who received oral semaglutide reported greater food craving control and higher mood scores compared to empagliflozin 25 mg [40]. This result might be associated with the fact that oral semaglutide at a dose of 14 mg compared to placebo decreased energy intake in patients with T2D [48]. More specifically, ad libitum total daily energy intake was reduced by 5096 kJ (a relative difference of 38.9%) in patients receiving oral semaglutide versus placebo in the lunch meal, evening meal, and evening snack boxes, resulting in dietary control and pronounced weight loss [48].

In summary, among patients spanning the continuum of T2D and with different background glucose-lowering medications, achieving a weight loss of at least 5% was greater with oral semaglutide 7 mg (19–27%) and 14 mg (30–44%) versus placebo (3–15%) and with oral semaglutide 14 mg (30–44%) versus liraglutide 1.8 mg (28%), empagliflozin 25 mg (36%), and sitagliptin 100 mg (10%) [49].

Table 2 is a compilation of all data above and considering the trial product as an estimand.

**Table 2:** Summary of the body weight reduction across the PIONEER trials considering the trial product without rescue medication (trial product estimand)

Trial	Treatment	Body weight (kg)					
		From baseline to week 26			From baseline to week 52		
		Mean change (kg)	EDT (95% CI) kg	p	Mean change (kg)	EDT (95% CI) kg	p
PIONEER 1	Sema 3 mg	− 1.7	− 0.2 (− 1.0 to 0.6)	0.71	–	–	–
	Sema 7 mg	− 2.5	− 1.0 (− 1.8 to − 0.2)	0.01	–	–	–
	Sema 14 mg	− 4.1	− 2.6 (− 3.4 to − 1.8)	< 0.001	–	–	–
	Pbo	− 1.5	–	–	–	–	–
PIONEER 2	Sema 14 mg	− 4.2	− 0.4 (− 1.0 to 1.0)	0.1358	− 4.7	− 0.9 (− 1.6 to − 0.2)	0.0114
	Empa 25 mg	− 3.8	–	–	− 3.8	–	–
PIONEER 3	Sema 3 mg	− 1.3	− 0.5 (− 1.0 to − 0.1)	0.03	− 1.7	− 0.7 (− 1.3 to − 0.1)	0.02
	Sema 7 mg	− 2.4	− 1.5 (− 2.0 to − 1.1)	< 0.001	− 2.5	− 1.5 (− 2.1 to − 0.9)	< 0.001
	Sema 14 mg	− 3.6	− 2.6 (− 3.1 to − 2.1)	< 0.001	− 4.2	− 2.9 (− 3.5 to − 2.3)	< 0.001
	Sita 100 mg	− 0.7	–	–	− 0.9	–	–
PIONEER 4	Sema 14 mg	− 4.7	− 1.5 (− 2.2 to − 0.9)^a^	< 0.0001^a^	− 5.0	− 1.8 (− 2.6 to − 1.0)^a^	< 0.0001^a^
			− 4.0 (− 4.8 to − 3.2)^b^	< 0.0001^b^		− 3.8 (− 4.8 to − 2.7)^b^	< 0.0001^b^
	Lira 1.8 mg	− 3.2	–	–	− 3.1	–	–
	Pbo	− 0.7	–	–	− 1.2	–	–
PIONEER 5	Sema 14 mg	− 3.7	− 2.7 (− 3.5 to − 1.9)	< 0.0001	–	–	–
	Pbo	− 1.1	–	–	–	–	–
PIONEER 7	Sema flex	–	–	–	− 2.9 (SE 0.3)	− 2.2 (− 2.9 to − 1.5)	< 0.0001
	Sita 100 mg	–	–	–	− 0.8 (SE 0.3)	–	–
PIONEER 8	Sema 3 mg	− 1.3	− 0.9 (− 1.6 to − 0.2)	0.0111	− 1.0	− 1.6 (− 2.6 to − 0.7)	0.0009
	Sema 7 mg	− 3.0	− 2.5 (− 3.2 to − 1.8)	< 0.0001	− 2.9	− 3.5 (− 4.5 to − 2.6)	< 0.0001
	Sema 14 mg	− 4.1	− 3.7 (− 4.4 to − 3.0)	< 0.0001	− 4.3	− 4.9 (− 5.9 to − 3.9)	< 0.0001
	Pbo	− 0.4	–	–	0.6	–	–

*CI* confidence interval; *Dula* dulaglutide; *EDT* estimated treatment differences; *Empa* empagliflozin; *Flex* flexible dose adjustment; *Lira* liraglutide; *Pbo* placebo; *SE* standard error; *Sema* oral semaglutide; *Sita* sitagliptin

^a^Versus liraglutide

^b^Versus placebo

## Oral semaglutide beyond glycemic control

### Efficacy and safety of oral semaglutide versus other GLP1-RA drugs

A systematic literature review used a network meta-analysis tool to calculate the relative efficacy and safety of once-daily oral semaglutide 14 mg versus dulaglutide 1.5 mg, twice-daily exenatide 10 μg, once-weekly exenatide 2 mg, once-daily liraglutide 1.8 mg, once-daily lixisenatide 20 μg, and once-weekly injectable semaglutide 0.5 and 1.0 mg (all GLP-1 RA class drugs) using seven clinical trials [50]. The results showed that oral semaglutide significantly reduced HbA1c levels if compared with dulaglutide (− 0.85%, 95% CI − 1.25 to − 0.45), exenatide [− 0.93%, 95% CI − 1.32 to − 0.54 (dose 10 μg) and − 0.89%, 95% CI − 1.27 to − 0.51 (dose 2 mg)], liraglutide (− 0.42%, 95% CI − 0.78 to − 0.05), and lixisenatide (− 1.32%, 95% CI − 1.74 to − 0.91) [50]. However, reductions in HbA1c were similar for oral and injectable semaglutide [− 0.32%, 95% CI − 0.72 to 0.08 (dose 0.5 mg) and 0.08%, 95% CI − 0.32 to 0.47 (dose 1.0 mg)] [50]. Oral semaglutide also significantly reduced weight compared to exenatide 2 mg (− 2.21 kg, 95% CI − 3.45 to − 0.92) and lixisenatide (− 2.39 kg, 95% CI − 3.66 to − 1.14) [50]. The other comparators were similar to oral semaglutide in bodyweight reduction [50]. According to safety, oral semaglutide provided similar odds of experiencing nausea and diarrhea compared with all other GLP-1 RA comparators [50]. On the other hand, the chance of vomiting was lower for oral semaglutide compared with dulaglutide [Odds ratios (OR) 0.03, 95% CI 0.01–0.73] and lixisenatide (OR 0.06, 95% CI 0.01–0.82) [50].

### Patient-reported outcomes

Good outcomes related to T2D therapy are not only measured by evaluating HbA1C levels. The multidisciplinary team must understand their patients to optimize each patient’s treatment experience. So, PIONEER 2, 4–8 measured patient-reported outcomes better understand the patient satisfaction with oral semaglutide and the impact of the treatment on well-being, quality of life, and weight-related quality of life compared with other active standard-of-care medications [40, 42, 43, 45, 46]. The results showed that oral semaglutide improved the feelings of unacceptably high blood sugars when compared with placebo or sitagliptin [42, 43, 45, 46]. Furthermore, when asked about craving control and craving for savory, patients also reported better feeling with oral semaglutide than empagliflozin [40]. The ETDs for general health and social functioning domains at week 26 significantly favored oral semaglutide over empagliflozin, whereas for role-physical and physical component summary domains at week 52 favored empagliflozin over oral semaglutide [40].

### Reduction of CV risk

CVD is the primary cause of death in patients with T2D, and new glucose-lowering therapies must show CV safety [51]. In the SUSTAIN-6 trial, the subcutaneous injection of semaglutide once a week reduced the relative risk of MACE by 26% compared to placebo [52]. In this trial, GLP-1 RA showed CV benefits on blood pressure, vascular endothelium, atherosclerosis progression and inflammation, and myocardial ischemia [52]. Furthermore, a meta-analysis that compiled data from the SUSTAIN-6 trial showed that CV benefits were associated with decreases in HbA1c and body weight [51].

PIONEER 6 assessed the CV outcomes of 14 mg of oral semaglutide in patients with T2D and high CV risk (defined as age  ≥  50 years with established CVD or CKD or age  ≥  60 years with CV risk factors only) [44]. This trial analyzed 3183 patients randomized to receive 14 mg of oral semaglutide or placebo, both in addition to standard-of-care treatment [44]. The primary endpoint was the time from randomization to the first occurrence of a MACE, a composite of death from CV causes (including undetermined causes of death), nonfatal myocardial infarction, or nonfatal stroke [44]. The secondary CV endpoints included the time from randomization to the first occurrence of an expanded composite outcome consisting of the primary endpoint plus unstable angina or heart failure, both resulting in hospitalization; or a composite of death from any cause, nonfatal myocardial infarction, or nonfatal stroke; and the individual components of these composite outcomes [44]. Furthermore, reductions in HbA1c, body weight, and lipid levels were measured, and adverse events were reported [44].

PIONEER 6 had an average duration of 15.9 months [44]. In this period, 99.7% of the selected patients completed the study, 84.7% in the oral semaglutide arm, and 90.1% in the placebo arm [44]. Furthermore, 75% of them received oral semaglutide or placebo for more than 12 months, and GI adverse events were the main reason for discontinuation [44]. The baseline characteristics of the patients were similar in both groups [44]. At the beginning of the study, some patients used metformin, insulin, sulfonylureas, or iSGLT2, in addition to antihypertensive, hypolipidemic, and antiplatelet or antithrombotic agents [44]. Interventions to initiate or intensify antidiabetic therapy were more frequent in the placebo group [44].

PIONEER 6 showed noninferiority of 14 mg of oral semaglutide compared to placebo related to CV events [44]. The primary outcome of a MACE occurred in 3.8% versus 4.8% of patients treated with oral semaglutide or placebo, respectively [hazard ratio (HR) 0.79, 95% CI 0.57–1.11; p  <  0.001] [44]. The outcomes from secondary endpoints showed that 0.9% versus 1.9% of patients died from CV causes (HR 0.49, 95% CI 0.27–0.92); 2.3% versus 1.9% had nonfatal myocardial infarction (HR 1.18, 95% CI 0.73–1.90); 0.8% versus 1.0% had nonfatal stroke (HR 0.74, 95% CI 0.35–1.57); and 1.4% versus 2.8% died from any cause (HR 0.51, 95% CI 0.31–0.84), all treated with oral semaglutide or placebo, respectively [44]. No unexpected adverse events were reported [44]. The main adverse events were associated with the gastrointestinal tract (11.6% oral semaglutide versus 6.5% placebo) and were responsible for discontinuing treatment in 6.8% of patients treated with oral semaglutide and 1.6% of those who received placebo [44]. Additionally, the levels of low-density lipoprotein cholesterol and triglycerides were modestly lower in the oral semaglutide group.

### Renal benefits of GLP-1 RA

One of the main causes of CKD and end-stage renal disease is T2D [53]. In addition, diabetic kidney disease is a major cause of morbidity and mortality in diabetes [53]. Kidney problems directly impact the choice of glycemic control medication in patients with CKD [53]. For example, metformin might need dose adjustment if the eGFR is  <  60 mL/min/1.73 m^2^ and is contraindicated if the eGFR is  <  30 mL/min/1.73 m^2^ [53, 54]. On the other hand, patients with kidney impairment presenting an eGFR of 15 ml/min/1.73 m^2^ theoretically can use some GLP-1 RAs, including subcutaneous semaglutide, once these drugs no longer need dose adjustment in these cases [53, 54].

A systematic review and meta-analysis compared kidney outcomes from seven trials with a total of 56,004 T2D participants treated with GLP-1 RA or placebo [55]. Lixisenatide (ELIXA trial), liraglutide (LEADER trial), subcutaneous semaglutide (SUSTAIN-6 trial), exenatide (EXSCEL trial), and dulaglutide (REWIND trial) were GLP-1 RA drugs evaluated for kidney outcomes in this meta-analysis [data for kidney events were not available for albiglutide (Harmony Outcomes) or oral semaglutide (PIONEER 6), so this analysis excluded the outcomes of these trials] [55]. Selected studies enrolled patients with recent acute coronary syndrome, stable cardiovascular disease, or cardiovascular risk factors [55]. After the period of the trial treatment, the main results showed that the worsening of glomerular filtration was similar in the GLP-1 RA group and the placebo group (HR 0.87, 95% CI 0.73–1.03; p  =  0.098), which indicated that these drugs did not affect kidney function [55]. Treatment with a GLP-1 receptor agonist reduced the broader composite kidney outcome [which consisted of the development of macroalbuminuria, worsening kidney function (doubling of serum creatinine or 40% or greater decline in eGFR), end-stage kidney disease, and kidney-related death] by 17% (HR 0.83, 95% CI 0.78–0.89), mainly due to a reduction in urinary albumin excretion [55].

The LEADER trial assessed a total of 9340 patients with T2D and high CV risk treated with semaglutide or placebo [56]. A secondary renal outcome of this trial was a composite of new-onset persistent macroalbuminuria, persistent doubling of the serum creatinine level, end-stage renal disease, or death due to renal disease [56]. The results showed that renal outcomes were less frequent in the liraglutide group than in the placebo group (HR 0.78, 95% CI, 0.67–0.92, p  =  0.003) [56]. New-onset persistent macroalbuminuria occurred in fewer patients in the liraglutide group (HR 0.74, 95% CI 0.60–0.91, p  =  0.004) [56]. Furthermore, in patients with an elevated renal risk (microalbuminuria or macroalbuminuria), a renal outcome also occurred in fewer patients treated with liraglutide (HR 0.81, 95% CI 0.68–0.96, p  =  0.02) [56]. A real-life study that better represents the everyday clinical practice of T2D patients evaluated a total of 38,731 individuals [57]. They were treated with a GLP-1 RA (liraglutide 92.5%, exenatide 6.2%, lixisenatide 0.7%, and dulaglutide 0.6%) or dipeptidyl peptidase 4 inhibitors [57]. Patients treated with GLP-1 RAs had a lower risk of serious renal events than those treated with dipeptidyl peptidase 4 inhibitors (HR 0.76, 95% CI 0.68–0.85) [57]. Additionally, GLP-1 RAs were associated with a significantly lower risk of renal replacement therapy (HR 0.73, 95% CI 0.62–0.87) and hospitalization for renal events (HR 0.73, 95% CI 0.65–0.83) [57].

In PIONEER 5, all eligible patients had moderate renal impairment, and overall, renal function was unchanged during the trial period in both groups once the median eGFR ratios (week 31 follow-up relative to baseline) were similar in both groups [HR 1.02, 95% CI 0.27–1.96 (oral semaglutide); HR 1.00, 95% CI 0.68–2.17 (placebo)] [43]. Furthermore, two patients in the oral semaglutide group had three nonserious events, and one patient in the placebo group had a nonserious adjudication committee-confirmed event of acute kidney injury and recovered [43].

Briefly, data about the effects of GLP-1 RAs on renal outcomes will be available [58]. The FLOW study is a randomized, double-blinded, parallel-group, placebo-controlled trial dedicated to clarifying the safety and impact of 1.0 mg of semaglutide weekly in people with renal impairment and T2D on major adverse renal events [58]. The study was initiated in June 2019 and will be completed after a 3- to 5-year follow-up with 3508 participants recruited [58]. The primary endpoint of the FLOW study is a renal composite measure of a persistent eGFR decline  ≥  50% from the start of the trial, end-stage renal disease, death from renal disease, or death from CVD [58]. Secondary outcome measures include renal outcomes, cardiovascular outcomes, changes in body weight, glycemic control, and blood pressure [58].

## The safety of oral semaglutide

The overall safety profile of oral semaglutide was similar in all PIONEER trials and was not unexpected, considering subcutaneous semaglutide and other GLP-1 RAs [39–46]. Adverse events related to oral semaglutide were mainly of mild or moderate severity and did not lead to permanent treatment interruption [39–46]. Premature discontinuation of the study drug due to adverse events was less than 15% in all studies and was more frequent in the oral semaglutide group than in the placebo or active standard-of-care group, mainly due to GI disorders (Table 3) [39–46]. The main adverse events reported in the PIONEER program were related to the GI tract, but of mild or moderate severity, and most were transient. Nausea (most common adverse event), vomiting, diarrhea, constipation, dyspepsia, and abdominal pain were the six most commonly reported events in the groups that received oral semaglutide compared to those who received placebo or comparators (Table 3) [39–46]. Adverse events were more common when the dose of oral semaglutide increased from 3 to 7 mg or from 7 to 14 mg [39–46].

**Table 3:** Summary of safety data across the PIONEER trials

Trial	Treatment (n)	Adverse events (n)		Discontinuation due to adverse events % (n)	Severe hypoglycemia episodes^b^ % (n)
		Total % (n)	Serious^a^ % (n)		
PIONEER 1	Sema 3 mg (175)	57.7 (101)	2.9 (5)	2.3 (4)	0
	Sema 7 mg (175)	53.1 (93)	1.7 (3)	4.0 (7)	0.6 (1)
	Sema 14 mg (175)	56.6 (99)	1.1 (2)	7.4 (13)	0
	Pbo (178)	56.6 (99)	4.5 (8)	2.2 (4)	0
PIONEER 2	Sema 14 mg (410)	70.5 (289)	6.6 (27)	10.7 (44)	1.7 (7)
	Empa 25 mg (409)	69.2 (283)	9.0 (37)	4.4 (18)	2.0 (8)
PIONEER 3	Sema 3 mg (466)	79.4 (370)	13.7 (64)	5.6 (26)	4.9 (23)
	Sema 7 mg (464)	78.2 (363)	10.1 (47)	5.8 (27)	5.2 (24)
	Sema 14 mg (465)	79.6 (370)	9.5 (44)	11.6 (54)	7.7 (36)
	Sita 100 mg (466)	83.3 (388)	12.4 (58)	5.2 (24)	8.4 (39)
PIONEER 4	Sema 14 mg (285)	80.0 (229)	11.0 (31)	11.0 (31)	1.0 (2)
	Lira 1.8 mg (284)	74.0 (211)	8.0 (22)	9.0 (26)	2.0 (7)
	Pbo (142)	67.0 (95)	11.0 (15)	4.0 (5)	2.0 (3)
PIONEER 5	Sema 14 mg (163)	75.0 (122)	12.0 (20)	15.0 (24)	6.0 (9)
	Pbo (161)	68.0 (109)	11.0 (18)	5.0 (8)	2.0 (3)
PIONEER 6	Sema 14 mg (1591)	–	18.9 (301)	11.6 (184)	1.4 (23)
	Pbo (1592)	–	22.5 (358)	6.5 (104)	0.8 (13)
PIONEER 7	Sema Flex (253)	78.0 (197)	9.0 (24)	9.0 (22)	0
	Sita 100 mg (250)	69.0 (172)	10.0 (24)	3.0 (8)	0
PIONEER 8	Sema 3 mg (184)	74.5 (137)	13.6 (25)	7.1 (13)	28.3 (52)
	Sema 7 mg (181)	78.5 (142)	10.5 (19)	8.8 (16)	26.0 (47)
	Sema 14 mg (181)	83.4 (151)	6.6 (12)	13.3 (24)	26.5 (48)
	Pbo (184)	75.5 (139)	9.2 (17)	2.7 (5)	29.3 (54)

*Dula* dulaglutide; *Empa* empagliflozin; *Flex* flexible dose adjustment; *GI* gastrointestinal; *Lira* liraglutide; *N* number; *Pbo* placebo; *Sema* oral semaglutide; *Sita* sitagliptin

^a^Serious adverse events are based on patient/event outcome or action criteria usually associated with events that pose a threat to the patient’s life or functioning

^b^Hypoglycemic episodes were considered severe and defined as blood glucose lower than 56 mg/dL (< 3.1 mmol/L) associated with symptoms consistent with hypoglycemia (defined according to the American Diabetes Association classification)

The use of oral semaglutide did not significantly decrease the rate of patients who completed the treatment [39–46]. In the PIONEER program, the mean treatment adherence of patients who received oral semaglutide 14 mg, liraglutide 1.8 mg, empagliflozin 25 mg, sitagliptin 100 mg and placebo were 82.6% (79.6–86.3%), 83.7%, 89.0%, 88.9% (86.9–90.8%), and 88.8% (87.6–90.1%) respectively [39–46]. GI reactions were the main cause of withdrew the study in oral semaglutide group [39–46]. Oral semaglutide intake demands ingestion care, such as fasting state and caution for water intake, which may decrease patient adherence [19]. Future real-world data will be necessary to realize if this ingestion care will impact oral semaglutide adherence. However, the adherence rate of injectable semaglutide in SUSTAIN trial was very similar to oral semaglutide (84.7% versus 82.6%, respectively) [31, 39–46]. Furthermore, the adherence of injectable and oral placebo was also similar in SUSTAIN versus PIONEER studies (89.7% versus 88.8%, respectively) [31, 39–46].

Severe hypoglycemic episodes occurred more frequently in patients receiving basal insulin in association with oral semaglutide (PIONEER 8) (Table 3) [46]. In general, a symptomatic decrease in blood glucose was uncommon (less than 8% in all groups that received oral semaglutide). In addition, all cases of hypoglycemia were considered mild or moderate [39–46, 59, 60].

Worsening of renal function was not reported in any of the studies, including PIONEER 5, where T2D patients had established CKD [39–46]. Acute pancreatitis and acute kidney injury were rare and were similar among patients who received oral semaglutide and comparators [39–46]. Although other GLP1-RA treatment studies showed some increase in lipase and amylase production, this observation was not associated with clinical manifestations of acute pancreatitis [39–46, 61].

Patients treated with oral semaglutide or liraglutide had a small decrease in blood pressure and a slight increase in heart rate compared to patients treated with non-GLP-1 RA comparators [39–46]. Malignant neoplasms and death were rare adverse events and occurred with equal or less frequency in the oral semaglutide group than in the placebo group [39–46]. Few deaths occurred in the PIONEER program, and all of them were judged by the investigators as unlikely to be related to the study drug [39–46].

## Benefits of early and effective treatment in T2D

Approximately 80% of the annual cost of T2D to the United Kingdom National Health Service arises from dealing with potentially avoidable long-term complications of T2D [62]. A direct therapeutic intervention aimed at controlling the patient’s glycemic costs at only 8% of the amount [62]. With this rationale, a study used the IMS Centre for Outcomes Research and Effectiveness (CORE) Diabetes Model (a nonproduct-specific diabetes policy analysis tool that performs real-time simulations developed by IMS Health, Danbury, CT, USA) to examine the impact of improved glycemic control in patients with T2D [62]. The results clearly show that modest improvements in glycemic control in T2D and sustained HbA1c control can significantly reduce the rates of diabetes-related microvascular complications and reduce foot ulcers, amputations, and neuropathy, which can reduce the current cost of T2D by 57% [62]. Besides decreasing the future treatments with comorbidities related to T2D, the adequate use of oral semaglutide 14 mg was considered a cost-effective treatment option versus empagliflozin 25 mg, sitagliptin 100 mg, and liraglutide 1.8 mg in the United Kingdom and the Netherlands [63, 64]. Based on long-term projections, diabetes-related complications were fewer with oral semaglutide of 14 mg, which yielded cost savings that partially offset its higher treatment costs versus empagliflozin and sitagliptin [63, 64]. Related to liraglutide, the treatment itself, even without considering costs with comorbidities, was lower with oral semaglutide [63, 64].

The importance of early and effective treatment was also discussed by Defronzo when he proposed that the T2D treatment algorithm must change [65]. Instead of a treatment that focuses on glucose control, the proposal goal was a therapy that should delay disease progression and eventual treatment failure based on the pathophysiology of T2D [65, 66]. The pathophysiologic abnormalities involved in glucose intolerance in T2D include (1) β-cell failure; (2) insulin resistance in muscle; (3) insulin resistance in the liver; (4) insulin resistance in adipocytes (increased lipolysis); (5) reduced incretin secretion and sensitivity (gastrointestinal); (6) increased glucagon secretion (α-cells); (7) enhanced glucose reabsorption (kidney); and (8) central nervous system insulin resistance resulting from neurotransmitter dysfunction (brain) [65, 66]. Together, these eight pathogenic mechanisms complete the T2D “ominous octet” [65, 66]. According to Defronzo, optimal management of T2D should include early initiation of therapy using multiple drugs, with different mechanisms of action, in combination [65]. However, oral semaglutide and other GLP-1 RA drugs act by increasing insulin secretion, inhibiting glucagon secretion that leads to reduced hepatic glucose production, correcting defects in the production of gastrointestinal incretins, and promoting an effect on the central nervous system that leads to appetite suppression and weight loss, which is reflected in increased muscle and liver sensitivity to insulin [67, 68]. That is, oral semaglutide corrects seven of the eight mechanisms involved in the pathophysiology of T2D [67].

## Conclusions

The innovative development of an oral formulation for the GLP-1 RA semaglutide was a major therapeutic advance for T2D. Phase II clinical trials and the PIONEER program thoroughly evaluated and proved the bioavailability, efficacy, and safety of oral semaglutide across the T2D spectrum. Different backgrounds of patients can use oral semaglutide, especially those who do not want to use injectable medication. More specifically, the PIONEER program showed that oral semaglutide is efficacy and safe for adults with early T2D managed by diet and exercise, adults with advanced disease requiring daily insulin, and adults with CVD and/or CKD. Furthermore, this drug was more effective than comparators for glycemic control and weight loss. The expectation is that the oral formulation will increase patient adherence to treatment, which is essential in controlling blood glucose and reducing complications and comorbidities. Comparisons between oral semaglutide and other medications not included in the PIONEER program, such as injectable semaglutide, could be considered a limitation of this study because they had different methodologies, dosages, and treatment times. In the future, Real-world data will be required to confirm if the outcomes seen in the PIONEER program are translated into clinical practice.

## Data Availability

Not applicable.

## Abbreviations

ADA: American Diabetes Association
ANVISA: Brazilian Health Regulatory Agency
BMI: Body mass index
CI: Confidence interval
CKD: Chronic kidney disease
CV: Cardiovascular
CVD: Cardiovascular disease
EASD: European Association for the Study of Diabetes
eGFR: Estimated glomerular filtration rate
ETD: Estimated treatment difference
FDA: Food and Drug Administration
GI: Gastrointestinal
GLP-1 RA: Glucagon-like peptide 1 receptor agonist
HbA1c: Glycated hemoglobin
HOMA-β: Homeostasis model assessment of β-cell function
HR: Hazard ratio
kg: Kilograms
kJ: Kilojoules
m: Meter
MACE: Major adverse cardiovascular event
mg: Milligrams
OR: Odds ratios
PIONEER: Peptide Innovation for Early Diabetes Treatment
SGLT2i: Sodium-glucose cotransporter 2 inhibitor
SNAC: Sodium N-[8-(2-hydroxybenzoyl) amino] caprylate
T2D: Type 2 diabetes
TZD: Thiazolidinedione
UKPDS: United Kingdom Prospective Diabetes Study
